# A box, a trough and marbles: How the Reed-Frost epidemic theory shaped epidemiological reasoning in the 20th century

**DOI:** 10.1007/s40656-021-00445-z

**Published:** 2021-08-30

**Authors:** Lukas Engelmann

**Affiliations:** University of Edinburgh, Edinburgh, UK

**Keywords:** Epidemiology, Epidemic theory, Modelling, History of science, Covid-19, Reed frost epidemic theory

## Abstract

The article takes the renewed popularity and interest in epidemiological modelling for Covid-19 as a point of departure to ask how modelling has historically shaped epidemiological reasoning. The focus lies on a particular model, developed in the late 1920s through a collaboration of the former field-epidemiologists and medical officer, Wade Hampton Frost, and the biostatistician and population ecologist Lowell Reed. Other than former approaches to epidemic theory in mathematical formula, the Reed-Frost epidemic theory was materialised in a simple mechanical analogue: a box with coloured marbles and a wooden trough. The article reconstructs how the introduction of this mechanical model has reshaped epidemiological reasoning by shifting the field from purely descriptive to analytical practices. It was not incidental that the history of this model coincided with the foundation of epidemiology as an academic discipline, as it valorised and institutionalised new theoretical contributions to the field. Through its versatility, the model shifted the field’s focus from mono-causal explanations informed by bacteriology, eugenics or sanitary perspectives towards the systematic consideration of epidemics as a set of interdependent and dynamic variables.

## Introduction

Much of the political response to COVID-19 in the UK—and in many other places—has rested on inferences derived from mathematical models. Projections, predictions and assumptions about the pandemic were made using models which assumed new prominence in public perceptions of disruptive science-led government policy. Modelled “projections enact a sense of control through evidencing,” (Rhodes et al., 2020) particularly in lieu of reliable empirical data and epistemic uncertainties (Daston, 2020). Models are not neutral, but have been accused to perpetuate their “built-in biopolitical assumptions”, which in turn reinforce concepts of herd-immunity (Hinchcliffe, 2020) or suggest interventions such as lockdowns (Caduff, 2020). This new prominence given to models as political tools in public life, as well as objects of civic concern might be attributed to the need for rapid decision-making within the uncertainties of pandemic disruption. However, ours is also a moment in which a long-nurtured and well-developed epistemic authority of modelling in epidemiological reasoning has become starkly visible. Models are not a new working object among epidemiologists and they have been used throughout most of the twentieth century to create, investigate, explore and teach controlled, simplified simulacra of the worlds of infectious diseases. The epistemic authority of modelling, as this article will demonstrate, sits indeed at the heart of the history of how epidemics were made complex and how in turn they gave rise to an academic and analytic form of epidemiological reasoning.

The sudden proliferation of models during the epidemiological urgency of COVID-19 has raised questions about validity, accuracy and reliability of inferences derived from modelling in pandemic policy. These questions are not new, and a rich literature on models and modelling has long grappled with the role of mathematical predictions in public health. Models have been shown to promote interdisciplinary frameworks as they are not contained by any single discipline (Morgan, 2012). Modelling substantiates a style of epidemiological practice, as Erika Mansnerus argues, that successfully incorporates interdependent factors, indicators and assumptions, such as the number of contacts of an individual, the probability of transmission or the likelihood of pre-existing immunity in exposed individuals (Mansnerus, 2015, p. 13). When transferred from the epidemiological research environment to inform policy, models combine disparate factors into a coherent story, shaping the chaos of a pandemic into an actionable entity (Opitz, 2017). Models not only simulate epidemics, but assume epistemic weight precisely through their performative capacity as “generative machines” (Bauer, 2013) to render epidemics into objects of knowledge that can be newly responded to and intervened in.

This article turns to the history of infectious disease modelling to ask what we might take away from the historical conditions under which models first became the workbenches of epidemiological theorizing. I focus on one particular example, developed in the 1920s and often referred to as the “Reed-Frost epidemic theory,” designed by the biostatistician Lowell Reed and the epidemiologists Wade Hampton Frost at Johns Hopkins University. As a set of mathematical assumptions about the changing ratio of susceptible and immune individuals in a population, the theory had much in common with earlier attempts of formalizing the dynamics of an epidemic. William Farr had famously plotted curves of smallpox outbreaks in the late nineteenth century; PD En'Ko in Russia arrived at a model in the 1890s, which could be fitted well to measles outbreaks, John Brownlee had approached the theory of epidemics through statistical studies of immunity, Hilda Hudson and Ronald Ross had already presented their calculations of an "a priori pathometry" in 1916 to the Royal Society in London, and Herbert Soper sought mathematical explanations for the periodicity of epidemic curves (Brownlee, 1906; Dietz, 1988; Flexner, 1922; Heesterbeek, 2005; Ross, 1916; Ross & Hudson, 1917; Soper, 1929). What sets the Reed-Frost theory apart, and what is likely also the reason it has been largely overlooked in the relevant historiography, is its materiality as an object. The model was designed and predominantly used as a “mechanical analogue,” (Fine, 1977) a dynamic model constructed out of an angled trough and a box of colored marbles, with which the dynamics of infectious diseases were visualized and taught.

The model’s materiality coupled with its status as a pedagogical instrument, I argue here, had decisive impact on how epidemics were newly theorized at the time. The mechanical form of this models is one, perhaps the crucial condition, to understand the emergent success of infectious disease modelling in the 1920s. The Reed-Frost epidemic theory asserted influence because it belonged to the kind of objects “that people grasped with their hands” (Chadarevian & Hopwood, 2004, p. 2) as it embodied and displayed a novel theoretical approach to teaching the dynamics of infectious diseases. Its novelty did, however, not only derive from its capacity to illustrate generalisable aspects in three dimensions. Comparable to physical models in economics or chemistry, its material structure also afforded a shift in theorizing (Morgan & Boumans, 2004). Where most contemporaneous formulas had been overly concerned with the calculation of populations ratios, this mechanical model moved the analytical focus to the question of “adequate contact” (Abbey, 1952), theorizing the problem of multiple and interdependent causation in epidemiological reasoning.

The following historical reconstruction of the mechanical analogue to the Reed-Frost epidemic theory will bring two historical aspects to bear on the present proliferation of covid-19 modelling. First, Reed and Frost’s mechanistic model is introduced as a significant and widely overlooked ancestor to the pervasive SIR modelling conventions in contemporary epidemiology.^1^ The astonishing versatility and resounding success of this original mechanistic model might be subject of anecdotal knowledge among some epidemiologist and infectious disease modellers (Lessler & Cummings, 2016; Merrell, 1976; Sartwell, 1976). However, I argue here that its historical position within the wider field of epidemiological reasoning remains still underestimated. The theoretical intervention embodied in the Reed-Frost model enabled epidemiologists to conceive of all kinds of epidemics—including of chronic diseases—as the effect of a multitude of causal factors. Models and the pedagogy of mechanistic modelling thus anticipated the “web of causation” that Brian MacMahon and Thomas Pugh introduced into epidemiology textbooks in 1971 (MacMahon & Pugh, 1971). Second, as a novel “working object” (Daston & Galison, 1992), models furnished epidemiology with a new analytical practice to sharpen its academic profile as a budding discipline. The collaboration of Reed and Frost integrated medical and bacteriological knowledge with approaches from population ecology, vital statistics, chemistry and natural history. In the combination of these perspectives, epidemics appeared no longer as simple mass effects of disease. Models offered their exploration and experimentation “sui generis” (Amsterdamska, 2005, p. 31), and framed them as phenomena that “arise from within” rather than to emerge de novo (Mendelsohn, 1998, p. 306).

Instead of following these theoretical and analytical trajectories over the second half of the twentieth century to the present—this will need to be subject of follow-on work—this article reconstructs some of the aspects with which the Reed-Frost epidemic theory secured its defining place in this history. The first section turns to the Reed-Frost epidemic theory itself to explore its deterministic mathematical notation as a differential equation. While the trajectory of modelling might not be representative for the wider field of epidemiology, particularly as its post-war focus shifted to non-communicable disease, this history holds significance for the development of an analytical tradition in the wider field. In the second section, I argue therefore that the rigidity of mathematical formalisation collided with the empirical virtues of Frost’s epidemiology and the dynamism of Reed’s population ecology and that the development of a mechanistic model dovetailed with their endeavour of establishing epidemiology as academic and analytical discipline. In the third section, I revisit the affordances of the box, trough and marbles to ask how this mechanical model shifted the focus of epidemiological considerations away from either bacteriological virulence or the susceptibility of host populations to enable instead a novel theorisation of “adequate contact” as function of epidemic dynamics.

## The Reed-Frost epidemic theory

Please do not apply too liberal any formulation of a law of epidemic. Such a simple scheme, as was given to illustrate the effect of mass-immunization, may be useful and in a sense correct—as indicating the kind of interplay—but must be taken only for what it is—and this applies very generally to rigid formalizations—they are diagrams rather than photographic reproductions.^2^

These words of warning were raised by Frost in the introduction to his lecture series *Epidemic Theory III* at Johns Hopkins School of Hygiene in the 1930s. The students—most likely studying public health—had just witnessed a "simple scheme" with which Frost visualized how the manipulation of an epidemic curve could be achieved through basic arithmetic steps. After a lengthy prologue on the complex interactions between hosts, pathogens and the environment, which epidemiologists had to grapple with when trying to understand the waxing and waning of an epidemic, Frost introduced a mechanical model to his students. In the lecture-hall, he used an angled trough and a box with approximately one hundred coloured marbles to demonstrate the essential dynamics of an epidemic. To simulate an outbreak, marbles were poured into the angled trough in a single file. The resulting colour pattern determined the ratio of infected, susceptible, and recovered individuals for a given time period. Before the marbles were returned to the box, infected were replaced with recovered marbles and susceptible marbles lying next to infected marbles became infected. Then, after randomization in the box, the next time period was poured into the trough.

This model, which Frost declared "useful and in a sense correct," simulated the probable series of events following from the introduction of an infectious agent into a fixed population of susceptible individuals within a confined space. The simple scheme exemplified "the kind of interplay" that was thought to govern an epidemic’s dynamic and emphasised possible effects of interventions used to alter the predicted course of an epidemic. The box as randomizer guaranteed a probabilistic dimension of the model, while the trough stood in for societal and environmental structures in which transmission might occur. These mechanical structures did not just serve the purpose of illustration and exemplification, but they did set the model apart from the formula it was based on.

In its written form, the Reed-Frost epidemic theory emerged as a deterministic formula, void of any randomness or probabilistic devices. The parameters of its algebraic expression were simple: the theory made no assumptions about the natural history of the disease, nor did it qualify the pathogen, the host and its environment.^3^ All infected individuals will be infectious to susceptible individuals within a time-bound period, while infected individuals will go on to develop immunity to the infection in the same period. Each individual has a fixed probability of coming into contact with other individuals within one time interval—a concept Reed and Frost had integrated from Soper’s work with reference to chemical laws of mass-action.^4^ Set up in this way, the theory was strictly deterministic, as all variables within such a model epidemic were determined by the model's logic. While the formula might be understood as a reasonable description "of the processes underlying outbreaks of acute infections within institutions (e.g. measles within high schools),” (Fine, 1977, p. 88) the deterministic theory was designed for mathematical clarity rather than to illuminate the dynamics of epidemics.

**Fig. 1.**
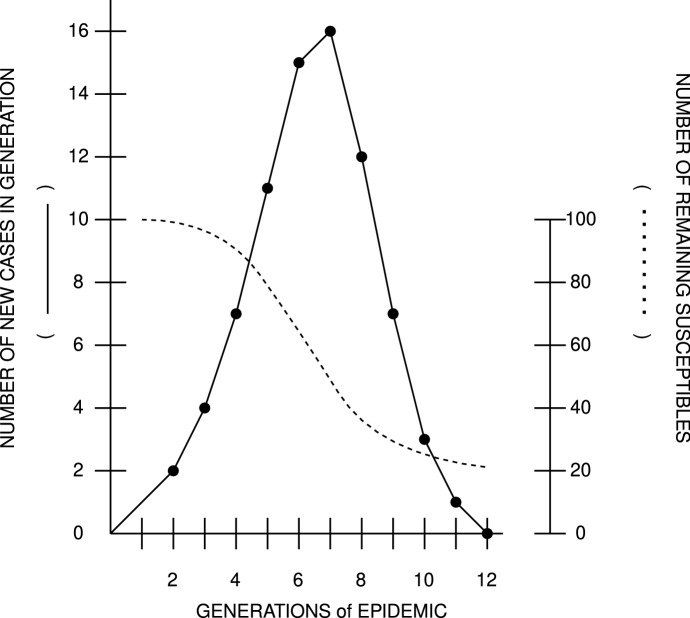
Graph of an epidemic curve according to the deterministic notation of the Reed-Frost epidemic theory. Here, one case was introduced into a population of 100 susceptible marbles. It was assumed that each individual had contact with two others during a time, with the graph in effect showing the development of the case numbers as well as the resulting number of remaining susceptible individuals in a population. Drawn after the original (Frost, 1976), with permission from Oxford University Press

The mathematical notation can be written out as the following differential equation: S^t^ and C^t^ are taken to be the numbers of susceptible individuals (S) and cases (C) during a time interval (t). Contact is noted as the probability (p), which assumes that a susceptible individual will come into contact with at least one case during a time period [*p* = (1−q)]. To predict the number of cases in an epidemic, the deterministic theory is mathematically expressed as follows: C^t^ + 1 = S^t^(1−qC^t^). The increasing number of cases in a time period (t + 1) is equal to the number of susceptible individuals divided by the probability for contact in that time period. This then gives an equation to express the withering of susceptible individuals in the same epidemic, with the assumption of acquired immunity: S^t^ + 1 = S^t^—C^t^ + 1. The shrinking number of S is equal to the given number of susceptible individuals, subtracting the number of cases growing over time. These two equations allow for a prediction and diagrammatic visualization of a simple theoretical epidemic. As the editor of the *American Journal of Epidemiology* visualized in a diagram in 1977, this deterministic model would always yield to the same curve, as all of its variables were ultimately determined by the model’s configuration (see Fig. 1).

**Fig. 2:**
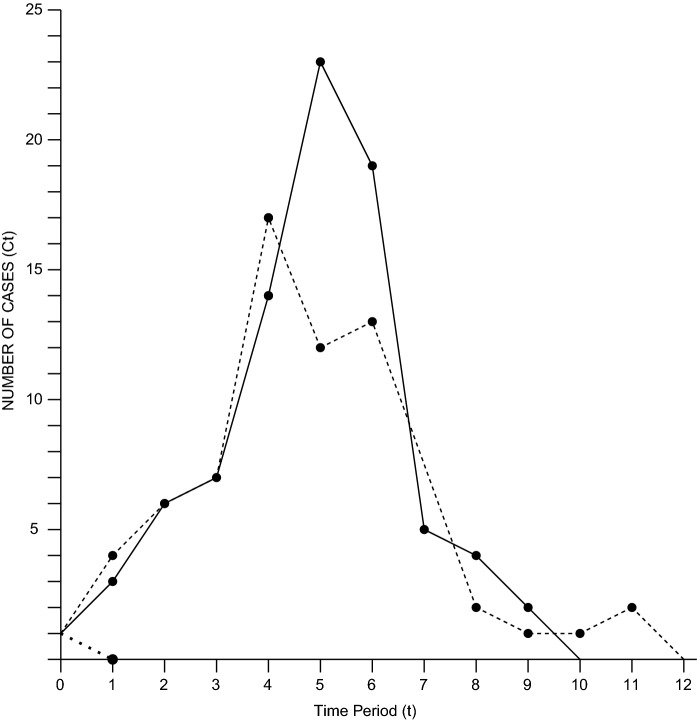
Graph to demonstrate the results of three epidemic simulations with the trough, box and marbles. Again, a single case was introduced into a population of 100 susceptible marbles. With one simulation to lead to only one case (……), the others lead to 73 (----) and 84 cases (–––). Drawn after the original (Fine, 1977), with permission from Oxford University Press

However, to make the model into a useful pedagogical instrument it needed to integrate a stochastic element by randomizing the numbers of susceptible individuals who could become infected. The notation gets reasonably complicated, and it might have been for that very reason that Reed and Frost decided in the late 1920s to build a mechanical model to demonstrate the stochastic version of their theory. The mechanical device consisted of marbles of four different colours in a trough: susceptible (S) were green, infected cases (C) were red, immune (I) were blue and blocks, or "contact neutralizers" (N), were white. Shaking the container with the marbles randomized the population after which they were poured into the trough in single file. In this row, individuals not separated by neutralizers were considered to have made sufficient contact, and susceptible marbles adjacent to infected marbles were now considered infected. This population of marbles was recorded, and susceptible marbles were replaced by infected marbles, while infected marbles were replaced by immune marbles. After randomization, the procedure was repeated until the epidemic expired. Multiple experiments with the same marbles led to different epidemic curves, as an element of chance now governed the model’s expression. Some iterations would immediately stall without any infection occurring, while others would closely follow the standard epidemic curves derived from the deterministic model. The model was versatile and allowed for experimental manipulations, as Fine emphasized in his appraisal. Immunization programs could be simulated by increasing the number of immunized marbles, multiple infections could be introduced to manipulate the curve and the amount of neutralizers (N) enabled the simulation of different rates of contact (see Fig. 2).

Archival sources reveal little with regards to any explicit considerations, which might have guided Reed’s and Frost’s thinking when designing the model. In his lectures, and on occasion elsewhere, Frost was a vocal sceptic about inferences made from schemes or models and then applied to real world epidemics. He asked his students to “note that any theory of epidemics which is sufficiently rigid and definite to be expressible in a mathematical statement is in all probability too rigid to be an exact representation of the phenomenon.”^5^ He wanted to make sure the model was not mistaken for a representation of an epidemic, nor did he expect his model to reveal new methods for forecasting or intervention. Importantly, to Frost, their model was not an instrument of prediction with which epidemiological research was to be elevated from painstaking work on the ground, in homes and communities affected by poliomyelitis, cholera or influenza. Instead, the model was for Frost an instrument to study the underlying dynamics of infectious diseases and to illustrate how interventions might impact outcomes.

Intriguingly, Frost had used the metaphor of a photograph to be entrusted with the representation of the complex event of a real epidemic. Only the mechanical reproduction of the framed picture could capture the varying shades of environmental conditions and the plethora of detail in which each case of infection has its own idiosyncratic coordinates and story. Only the camera could, to invoke Frost's contemporary Walter Benjamin, afford a sense of "the immense and unexpected field of action" (Benjamin, 1969, p. 236) that any event, and perhaps even more so, any epidemic crisis brought. The diagram, compared to the photograph, conveyed a set of previously considered relations to communicate a simplified illustration of selected components from the natural world: here, questions of relations, directions, and iterations were prioritized over quantity of detail (Engelmann et al., 2019). With the comparison, Frost reinforced the well-established separation between empirical and theoretical work, between observation in epidemic-stricken locales and scholars’ work with models, paper technologies and tools, diagrams and equations (Kaiser, 2009; Klein, 2003; MacKenzie, 2018; Morgan, 2012). The model, Reed and Frost had developed, was supposed to exemplify the relations and connections that seem to structure outbreaks, but it should not replace nor question the value of empirical observation.

Importantly, the Reed-Frost model was designed as a teaching instrument and neither Reed nor Frost considered a dedicated publication of the model and its underlying theory to be of any value. Frost offered an outline of the model’s reasoning in a 1928 lecture at Harvard, which was published posthumously in 1976. In the lecture, which offers some key considerations guiding Frost’s scientific approach to epidemiology, he contemplated the possibility of fitting epidemiological knowledge into general theories, which could be made vivid in the classroom through mechanical models (Frost, 1976, p. 142). In line with this pedagogical reasoning, the Reed-Frost model was a useful, and perhaps particularly successful instrument to teach fundamental principles of their epidemiological theory. "It might even be argued," Fine suggests in his review of the Reed-Frost model, that it was through classroom teaching "that models have had their greatest impact upon the practice of epidemiology today." (Fine, 1977, p. 87)

**Fig. 3:**
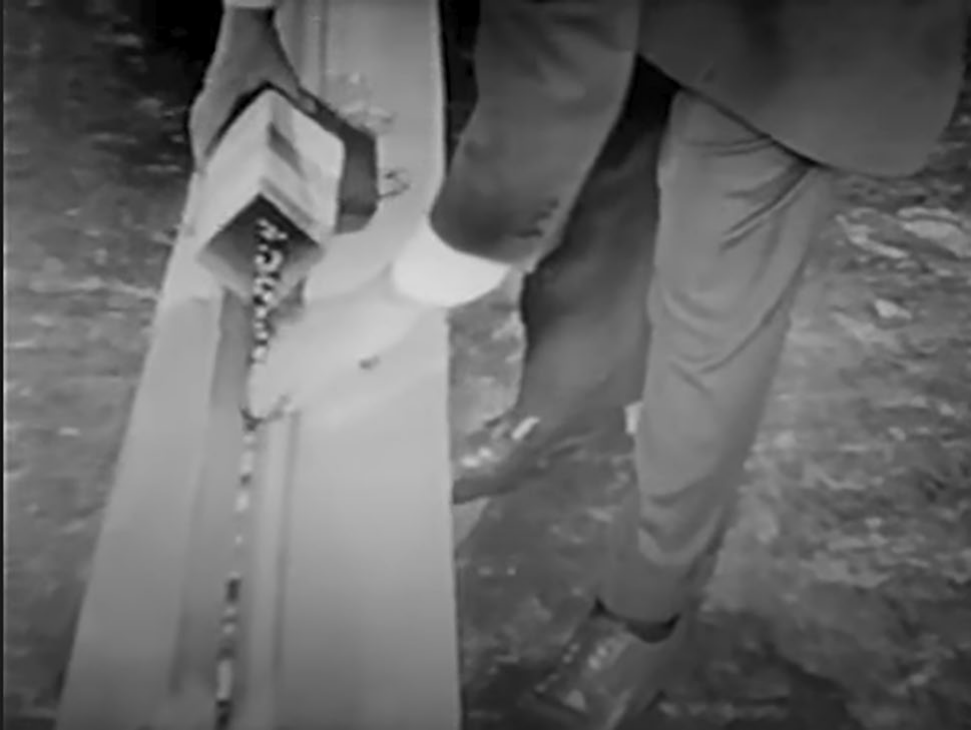
Still from “The Johns Hopkins Science Review” about the Reed-Frost epidemic theory from 1951. Here, Lowell Reed demonstrates the model's use, pouring marbles into the trough to simulate a time period of an epidemic. “Epidemic theory—what is it?,” Johns Hopkins Television Programs 1948–1960, Special Collections Milton S. Eisenhower Library, The Johns Hopkins University. With permission from Special Collections of the Milton Eisenhower Library at Johns Hopkins University

Only after the model had developed a life of its own, being used by other epidemiologists, replicated, and celebrated in epidemiological lectures across the US and in the UK, Reed expounded on its purpose and value explicitly (Frost had already died in 1938). In 1951, Reed presented a segment for the TV production "The Johns Hopkins Science Review", in which he explained to a public audience the merits of epidemic theory with his model. By 1951 Reed had placed the model at the heart of how he conceived of the scientific method in epidemiological research. He described and demonstrated how the marbles rolling down the trough is the “workbench of the epidemiologist's laboratory,” and called the mechanical construction “a critical instrument for experimental epidemiological science.”^6^ To emphasize the field’s value to the public, and to underline the rigor with which epidemiology pursued the protection of the nation’s health, Reed used the model to demonstrate what epidemiological scientists really do when they do epidemiology (See Fig. 3).

The models functions, its mechanical construction, the combination of the probabilistic box with the linear trough and the seriality of time-periods implied by its usage were supposed to reshape how causes were conceptualised, while shifting the analytical focus to the multiple conditions under which the dynamics of an outbreak unfold. In this way, Reed and Frost’s model was perhaps supposed to do for epidemiology what Feynman's infamous diagram had done for theoretical physics. As a drastic simplification of complex and lengthy calculations, Feynman’s diagrams had been dispersed, were used in classrooms, lecture halls and seminars, while being re-drawn, re-used and re-interpreted in pedagogical settings. In Kaiser’s interpretation of these diagrams as important paper tools for the theoretical practice in physics, they do not—as Latour would argue—appear as instruments of cohesion, consistency and immutability (Latour, 1990), but were subject to unfolding variations in their representation, in their application and in their interpretation (Kaiser, 2009, p. 7). Similar to these diagrams, Reed and Frost’s model lent itself to dismantle causal concepts, prevalent in epidemiological thinking and it was the mutability of the model, that allowed it to shape theoretical foundations for epidemiology’s elevation into the realms of academic disciplines.

## Disciplining epidemiology

In 1919, when Frost took up the chair of the world’s first dedicated department of epidemiology, the field was structured by extensive discussions about its novel shape as an academic discipline. From the outset, Frost acknowledged that the field was only "provisionally defined" and its shape "has not been covered in a systematic manner."^7^ Epidemiological chairs had been established in medical schools elsewhere, while epidemiological societies and clubs had advanced epidemiological thinking in a more or less institutionalized way, particularly in the UK (Amsterdamska, 2005; Magnello & Hardy, 2002; Steere-Williams, 2020). But setting up the department of epidemiology at the Hopkins School for Hygiene required a systematic consideration of the field to vouch for its unique scientific contribution and to demarcate its institutional relationship to the existing academic landscape.

In his new position as head of department, Frost sought to establish an empirical research field dedicated to the “study of infectious disease in nature.”(Frost, 1941, p. 504) Particularly the study of infections without signs of disease or illness, Frost wrote in 1928, was supposed to "prepare our minds for clearer interpretations of the more obscure epidemiology of other diseases."(Frost, 1976, p. 144) In parallel, Frost sought to set his epidemiological research on firm, but novel scientific grounds. His teaching and lectures were regularly prefaced with a friendly reminder about the first principles of epidemiology as a positive science. With reference to Karl Pearson, he defined epidemiological research as dedicated to "the classification of facts, the recognition of their sequence and relative significance, and the habit of forming a judgment upon these facts unbiased by personal feeling."^8^ He invoked Pierre-Eugène-Marcellin Berthelot to remind students that the purpose of such a science was to establish facts and put them into immediate relations, rather than to invest in the understanding of a cause or end. The methods Frost taught, and the laboratory studies he led, were exercises in problem-led collection, analysis and communication of data. According to his students, Frost’s was an "example of painstaking, systematic and scholarly approach to problems."(Sartwell, 1976) However, as epidemiology was “never, in fact, developed, as a purely descriptive science,” but rather an endeavour to enable a novel understanding of disease, its distribution, and control, it would require “a theory or philosophy of disease.”(Frost, 1941, p. 497) The question was, how this theoretical work would set epidemiology on discrete and sufficiently unique grounds.

Fee characterized Frost’s agenda as an attempt to craft a field of applied public health science. Exemplified by Frost's own biography as a field epidemiologist, this was an effort to maintain a practice of epidemiology based in experience, which could be useful to medical officers in their field investigations, while still enabling "theoretical development."(Fee, 2016, p. 133) Frost himself defined the purpose of the new department with three distinct goals: first, students were to learn about "certain fundamental principles governing the occurrence of infectious diseases"; second, they would become familiar with "the special methods applicable to study in this field"; and third, they should understand how to apply these principles and methods to "public health administration."^9^

In line with these pedagogic principles, Frost’s teaching method focused on case studies. The department's portfolio included lecturing on the diagnosis of discrete epidemics using data from historical events, the alignment of experimental data with knowledge about aetiologies of infectious diseases, and an introduction to using epidemiological records in the "administrative guidance in the control of infectious diseases."^10^ The lectures, which according to Fee enjoyed great popularity among students, were accompanied by laboratory experiments as well as field studies (Fee, 2016, p. 134). Medical officers-in-training were encouraged to study data collected from ongoing outbreaks of poliomyelitis or the common cold, to apply analytical instruments, to develop strategies of prevention and containment, and to learn best practice of statistical methods. Each student developed fieldwork with the local authorities in Baltimore, using case data about prevalent infectious diseases while conducting sanitary surveys within the city. Frost stressed from the outset that a successful epidemiological department would rely on extensive ties with institutions outside of the university, as well as on strong collaboration across the university and especially with the Johns Hopkins Hospital. Such collaboration was not merely an effort to prevent duplication of "effort and expense, but more largely because of a sincere belief in the principle of interdepartmental cooperation."^11^

Many historians have focused on the development of epidemiology in the aftermath of the success of the medical sciences in the late nineteenth century, particularly with regards to bacteriology. Frost, like many of his contemporaries, thought of the relationship between pathogens and hosts as one of “seeds and soil,”^12^ emphasising, as Worboys suggested for the development of germ theories at large, their mutual dependency (Worboys 2000). However, Frost thought of this binary relationship within the context of environmental factors, which might determine susceptibility of the host (soil) and impact on the virulence of the pathogen (seed). Anticipating, what post-war epidemiologists began to frame as the epidemiological triad, Frost’s conceptualisation of epidemiological reasoning was strictly dedicated to the interrelations of agent, host and environment. He was acutely aware of the potential narrowing of the disciplines scope, as bacteriology’s “powerful new methods of identifying the causes of diseases” threatened to shift the focus away from more complex explanatory models and invited a new concern with specific pathogens and their control in epidemiology (Fee, 2016). A programmatic statement of a conference on Epidemiology, organised by Frost at Johns Hopkins University in 1927 defined thus the “science of epidemiology” to be concerned with the “natural history of disease as it is expressed in groups of persons related by some common factor of age, sex, race, location, or occupation as distinct from the development of disease in individuals.” Contributing to the understanding of the mode of distribution, the aetiology, possible treatment and practices of prevention, would require the “application of at least the following associated sciences and arts. Clinical and laboratory diagnosis: (the practice of medicine and public health, bacteriology and immunity), Collection, tabulation, and analysis of morbidity and mortality reports: (Vital statistics, demography, biometry), Correlation of external environment (sanitary science), and internal environment (Parasitology) of man with the development and prevention of disease.”^13^ It was Frost’s explicit ambition to establish epidemiology as a nothing less but a generalist science.

Krieger's comprehensive historical overview of the field acknowledges its intersections with other scientific developments, as epidemiologists grappled with the “welter of epidemiology, clinical and laboratory evidence about infectious diseases." (Krieger, 2011, p. 97) To combine these fields and to apply them evenly to the inference of epidemiological knowledge was not always successful. Some epidemiologists appeared to be overly invested in the translation of biological principles into the more complex dynamic of infectious diseases, others remained attached to eugenics and its focus on how societal conditions, particularly the economic status of individuals, shape health outcomes (Krieger, 2012, p. 647). Heesterbeck describes a schism in the field since the late nineteenth century, as causal theories were either derived from the infective power of the organism or from the susceptibility of a population (Heesterbeek, 2005, p. 85). The question was, how to define the field's specific scientific status, if it were not aligned as a secondary science to either the authority of the bacteriological laboratory or to spurious claims about population fitness (Parascandola, 1998).

Amsterdamska has shown for the UK that epidemiologists between 1890 and World War I remained indeed "relatively uninterested" in the epistemic offerings of the laboratory (Amsterdamska, 2005, p. 21). Bacteriology did not offer deep insights into most of the questions epidemiologists were asking. The identification of a pathogen did not illuminate the structure of prevalence rates, nor did it allow inferences about the spaces and locales and the social conditions of outbreaks. However, the field’s “continuous involvement with the politics and practice of public health” remained also an obstacle for its scientific identity (Amsterdamska, 2005, p. 18). To break the impasse, some epidemiologists, like John Brownlee, warmed to Pasteur’s suggestion of variable virulence to explain the waxing and waning of epidemic dynamics (Amsterdamska, 2001, p. 140). However, many epidemiologists safeguarded their discrete perspectives by turning instead to mathematical theorising to develop causal theories focused predominantly on the changing composition of populations.

As some have emphasized since Hacking's *The Taming of Chance*, medical statistics offered the strongest set of tools with which to develop a novel and discrete scientific identity for the project of an academic epidemiology (Hacking, 1990). According to Matthews, statistics could contribute a strong "scientific self-image", as epidemiology expanded from empirical social science to form a strong body of formal analytical techniques and methods (Matthews, 1995, p. 86). Hilts emphasises that particularly the "biometricians gave to epidemiology a new mathematical rigor and the language of correlation coefficients, skew curves, and tests for the goodness of fit." (Hilts, 1980, p. 50) Spearheaded by Karl Pearson and translated into British epidemiology by Major Greenwood, biometricians forcefully argued for the almost universal application of statistical methods. Rather than just a tool to improve standards in clinical research or the bacteriological laboratory, Pearson and his collaborators sought to establish statistics as an authoritative method of scientific inference (Porter, 1995). In particular, the application of statistical analysis to the study of life was to bring biological insights that neither the laboratory nor the physiological experiment could offer. Greenwood, in a letter to Pearson, emphasized his conviction that indeed the laboratory and its established methods should not have any validity if results were not also confirmed through statistical methods:

The fact of the matter is that we are standing at the parting of the ways in medicine. The day is gone by when purely experimental work in either physiology or pathology can greatly advance knowledge. In the days of Ludwig, Claude Bernard and Pasteur the field was comparatively open; it is so no longer and the need for more rigorous logic and statistical methods of analysis must be realised sooner or later even by the average consultant (Greenwood, quoted in Matthews, 1995, p. 105).

Fortifying his own belief that epidemiological laws should be discerned and formalized with mathematical accuracy, Pearson gave up quickly to convince the stubborn London epidemiologists of his political rationality.^14^ He focused instead on training dedicated "iatro-mathematicians", whose role was to redress medical and epidemiological questions to the perspectives of biometrics, once again prioritizing the consideration of population dynamics over environmental or bacteriological approaches (Hilts, 1980, p. 50). One of his dedicated students, among whom were renowned epidemiologists like Greenwood and Soper, was Raymond Pearl.

In 1917, Pearl brought Pearson’s school of thought to the US to establish population ecology in North America (Kingsland, 2005). Pearl was invited by Welch to take up a new chair in vital statistics at Johns Hopkins, to elevate statistics from an administrative practice to an innovative research field within the School of Hygiene. As Fee emphasizes, Pearl, like Pearson, was eager to rethink the study of biology on a larger scale, integrating the analysis of populations into the understanding of life and death (Fee, 2016, p. 137). When Pearl joined Hopkins, he brought with him Lowell Reed, a tactful and discrete mathematician. While Pearl was famous for overbearing generalizations about the capacity of vital statistics and sported interests across the sciences and humanities, Reed was a specialists, as well as a "superb teacher", championing laboratory methods in statistical training (Fee, 2016, p. 139). Eventually, Reed succeeded in establishing a department for biometry and vital statistics in 1924, which in association with his chair set out to focus on the mathematical analysis of data about human health and disease (Fee, 2016, p. 143).

By the mid-1920s, Reed had already made his name as an author of a series of mathematical theories. His name was attached to the Reed-Mettell method used to develop abridged life tables, as well as the Reed-Muench method, used to determine lethal dosages and Reed had made substantial contributions to the logistic curve as explanation for population growth. The biometrician, who was made full professor in 1925, had become an eminent figure in the formalization of population dynamics when he ventured into the field of public health and epidemiology (Kiser, 1966). Reed and Frost’s collaboration began formally in 1928 with a joint grant from the School of Hygiene and the School of Medicine to study the common cold and its relation to influenza. However, they had been close colleagues, perhaps friends, since as early as 1923, Fee reckoned (Fee, 2016, p. 138).

Several propositions that would later re-emerge in epidemiological modelling had been foundational to the field of population ecology, which both Pearl and Reed had advanced in the 1910s and 1920s. Their work on the logistic curve as a description of human and animal population growth constituted a formal and conceptual precedent for the model Reed and Frost put forward a few years later. Under the assumption that populations grow exponentially until reaching a limit due to lack of crucial resources, Pearl and Reed assumed—like Verhulst had earlier—that “the growth of a population decreases in linear fashion with the density of a population.”(Kingsland, 1982, p. 32) Although the logistic curve had been fitted on American census records, Kingsland argued that the resulting graph was not an empirical statement, but rather the expression of a theoretical law—in the sense of Pearson’s statistical laws—of population growth. It was widely seen as radical proposition. However, the open question was to what extent these postulated laws about population growth incorporated phenomena like epidemics (Pearl & Reed, 1920). Were epidemics external factors that sustained deviations from the postulated regularity of the logistic curve, or were epidemics governed by similar principles and statistical laws and thus part of the equation?

An institutional association at Johns Hopkins between population ecology and epidemiology at that time did not come easily. The biometricians in population ecology were known for formulaic approaches to data, eager to discern laws and patterns within large sets of birth and mortality statistics, census data and hospital records. Biometricians developed methods to infer insights from ever-larger datasets, to reject and revise dogma and standing hypotheses. Davenport’s studies on feeble-mindedness in the US, as Porter has recently shown, pioneered such transformative inferences from larger datasets (Porter, 2018, p. 217 ff.). While biometricians had focused predominantly on the dynamics of populations, it would be a mistake to assume that epidemiologists like Frost had retreated to mono-causal explanations delivered by bacteriology. It would be another mistake to assume that the collaboration of Reed and Frost in the development of their model had led to the integration of epidemiology into the field of vital statistics—as Pearson had wished for. Rather, Frost in his definition of epidemiology referred to “quantitative epidemiological descriptions” that are as important for the understanding of an epidemic, as were fine-grained observations and descriptions of “local environment, personal habits, past history, and individual traits.”(Frost, 1941, p. 496).

Returning to Frost’s analogy of the relation between theory and descriptions as one associated with the diagram and the photograph, the development of the Reed-Frost epidemic theory encapsulated a rather subtle, but important epistemological shift. The collaboration of the biometrician Reed and the predominantly empirically orientated epidemiologist, Frost, would effectively turn a descriptive practice into an analytical science. Without disavowing the value of observation and descriptions, Frost conceded that epidemiology could not be a “purely descriptive science.” But to advance epidemiological theory and to develop a field that not only counts and maps cases but contributes to the understanding of a disease’s “nature, sources, means of spread, and eventually its control,” it was important to overcome the persistence of simplistic causal theories and to replace them with analytical approaches (Frost, 1941, p. 497).

In line with this approach, the Reed-Frost epidemic theory was geared towards the correlation of interdependent variables, rather than to investigate single culprits for an epidemic outbreak. The mechanical model embodied a departure from decades of deterministic haggling between explanatory models, where bacteriologists had prioritised the pathogen, where vital statisticians—like Reed—had focused on populations and where traditional sanitarians and field epidemiologists—like Frost—remained concerned with the environment. Reed and Frost’s mechanized “diagram” found its central purpose in drawing apart persisting causal theories, merging research conventions and disciplinary approaches, while offering a versatile teaching instrument to develop a new practice of analytic epidemic theory.

## Making a model science

As Fine reports in the 1970s, the model quickly became a staple in discussions among epidemiologists in the 1940s. It was adapted and developed in departments across the US, and was the subject of a long series of interpretative, extending and revising publications (Abbey, 1952; Fine, 1977). Some recent publication continue to position the model as the origin of a history of “mechanistic modelling” in public health (Lessler & Cummings, 2016; Phillips, 2021). Without a doubt, the Reed-Frost epidemic theory became a cornerstone for a budding tradition of building models in epidemiology since the 1920s. In its mechanical simplicity, equipped just with a box, a trough, and coloured marbles the model dissolved disciplinary boundaries, while offering a new way to theorize the dynamics of infectious diseases.

Morgan reminds us in her history of modelling in economics that modelling and mathematical proofing are different styles of reasoning. Morgan considers the making of models a discrete epistemic genre, "a practical mode of reasoning to gain knowledge about the economic world."(Morgan, 2012, p. 18) As such, in economics—as well as in epidemiology—a style of reasoning dedicated to modelling has proliferated with the support of mathematical approaches, but should not be seen as identical with it. Where the mathematical formula postulated and described general laws of epidemic distribution, making a physical model enabled a different approach to the exploration and experimental denotation of aspects of epidemiology. Morgan hence suggests a strong focus on the modalities of model objects to comprehend how the mechanical construction of models and working with physical models might have shaped scientific practice.

Making models requires different degrees of formalisation. Reed and Frost developed a mathematical expression for the characteristics they inferred from vital statistics (Reed) and which had been observed in epidemics on the ground (Frost). Abbey assumes their formula to have been heavily influenced by Soper’s work. In the early 1920s, Soper postulated a hypothetical community in which susceptibility to disease and the capacity to transmit disease was of equal power to all individuals and delivered a mathematical equation which fitted roughly to real-world measles outbreaks (Soper, 1929). Soper assumed that the transmission of disease within a population ultimately followed a similar dynamics as “the law of mass-actions” among chemical molecules, according to which “the numbers of cases infected by one case is proportional to the number of susceptibles in the community.”(Soper, cited in Heesterbeek, 2005, p. 97) As Abbey points out, Reed and Frost took issue with the assumption of a perpetual flow of cases, governed by the number of susceptible individuals and considered this to be a critical oversight in Soper’s equation. Their question was, how could the dynamic of an epidemic be modelled so that it took into account more than the changing ratios of a population, of the distribution of infectious, susceptible and immune individuals? Moving beyond the population as the predominant focus of mathematical approaches to epidemiological phenomena, their model was supposed to allow for experimental engagement with the factors that contributed for an infection to occur in between individuals. They turned thus to the question of contact.

Reed and Frost had designed their model first of all to allow for active cases to only infect susceptible individuals which were in direct contact within a time period. To achieve this modification, they turned to the urn and marbles, the standard modelling inventory of probabilistic reasoning since Bernoulli (Hacking, 1990, p. 101). In its basic form it was used to infer the probability of a ratio of differently coloured marbles based on a variable number of drawings from the urn. Using and adapting this mechanical instrument emphasised that all epidemics are to some extent governed by elements of chance. Reed and Frost, however, used the urn as a randomiser to then simulate the ongoing outbreak in the trough. While the marbles continued to represent the population and its changing ratio in an epidemic, the trough assumed the position of a time-period in which the marbles come into a structured contact—a single file—with each other.

Through the use of such coloured marbles, Krieger argues, a specific statistical understanding of population has come to be established in epidemiological reasoning. The hypothetical population of marbles in a box is void of intrinsic relations and disavows inferences between individual risks and population patterns. While such models might determine chances for individuals to catch a disease, it cannot ever determine which individual, or marble, will become a case. However, a physical model, so Krieger argues, still affords the epidemiologist a valuable visualisation of “structured chances.” (Krieger, 2012) Combining an instrument of randomization, such as the box in which the marbles are mixed, with a trough, in which the marbles form a pattern, enhanced an understanding of epidemics as phenomena shaped both by the changing ratio of a population as well as by structures that arise from multiple causal processes governing infection.

Devices like the mechanical analogue to the Reed-Frost epidemic theory therefore forcefully demonstrated that neither the innate qualities found in a population or the specific capacities of a pathogen were assumed to govern epidemics, but that the structural—or environmental—forces governing their interaction were just as significant. As Abbey in her commentary on the model has pointed out, a key advantage of the mechanical analogue was to prioritise the question of “adequate contact.” The models was predicated on the assumption that only susceptible individuals coming into contact with a case would yield to an infection. Susceptible marbles lying before and after an infected marble would become infected. Their status would last for one time period before the individual acquired immunity. For all susceptible individuals in the marble population there is thus a dynamic probability of “coming into adequate contact” with an infected marble, depending on where each marble lands in the single file in the trough within one time period. “Adequate contact” was thus understood as a stand-in for all possible factors determining the actual transmission of an infectious disease within a population. Abbey argued:

The probability of contact, in this sense, depends on the susceptibility or resistance of the host, the infectivity of the parasite, the length of exposure and size of dose necessary to produce the disease, as well as the environmental conditions for the transfer of the organism. (Abbey, 1952, p. 205)

From Morgan's work, it follows that a model, like the result of the Reed-Frost collaboration, allows for development of an experimental scientific inquiry that considers not only isolated aspects of epidemiological problems, but aims to contribute to the development of general theory. As the model becomes itself a representation of the principles that undergird epidemics, it begins to constitute what an epidemic ought to be. A general theory not only explains individual outbreaks to answer questions of the who, what and when of a specific disease in a particular location. Rather, it introduces the hypothetical epidemic as an object of enquiry, or—with Daston and Galison as a “working object,”—around which the practice of epidemiological research is assembled and from which the world of epidemics can be envisioned. How then did the Reed-Frost epidemic theory define what epidemics are and how epidemiology was supposed to study them?

In 1928, while lecturing at Harvard, Frost had offered further background to the ideas, which led to the collaborative development of the model. Firstly, developing a new general theory required rejecting that "epidemics originated de novo*.*"(Frost, 1976, p. 141) Since specific infectious pathogens had been identified it was no longer tenable to assume that each epidemic outbreak bore no resemblance to any previous one. This acknowledgement resulted in a new quest to identify the factors for the waxing and waning of epidemics, and to explain why epidemics seem to follow rhythms similar to the development of certain animal populations. The first explanation, many turned to across the nineteenth century, assumed that "prevalence of an infectious disease was strictly proportionate to the chance of contact." Epidemic spread was attributed in principle to the environment and to circumstances favouring the distribution of a pathogen (Frost, 1976, p. 141). Second, many bacteriologists had long argued that dynamics of epidemics adhered to the variability in the infective "properties of specific microorganisms," replacing older views that considered infectivity to be an inflexible characteristic of bacteria (Frost, 1976, p. 143). Third, the susceptibility of host organisms, as detailed by immunologists, were also subject to variation in relation to a range of factors, some directly connected to previous infections or to their habitat (for example in tropical diseases). Frost wrote:

The known differences between different diseases as regards variability of the specific microorganism, its period of survival in the individual host, the ratio of subclinical to clinical infections, the character and distribution of natural host resistance, the degree and durability of acquired immunity, and the kind of conditions necessary for conveyance from host to host—all these are sufficient to account for the widest differences in periodicity and range of epidemics in different diseases; and we can hardly expect to discover any simple and general law which will take account of all these variables. (Frost, 1976, p. 151)

According to Frost in 1928, none of the traditional building blocks of epidemiological arguments, not the environment, nor variable virulence in pathogens or the vulnerability of populations offered a stable foundation to draw causal inferences on the dynamics of epidemics. Frost’s elaborations suggest a conceptual gestation of what MacMahon and Pugh would frame in post-war epidemiology as the epidemiological triad: while agent, host and environment were implicated in the dynamics of an epidemic, causal inferences could no longer meaningful attributed to one of these building blocks of epidemiological reasoning. Any inference needed to consider the web of causation, in which each factor might impact on each other one (MacMahon & Pugh, 1971).

One key observation by Frost was that complex interdependencies seemed to be at work among these three discrete factors: environmental aspects might have influence on the infectivity of the pathogen and the susceptibility of the host; population behaviour could change the intensity of environmental circumstances; and passage through host bodies affected the virulence of infectious agents. Frost concluded that:

Each of these factors is subject to many and complex variations, which we can recognize as possibilities, and which we may demonstrate as realities, but which we can measure only imperfectly under natural conditions with our present means of observation. (Frost, 1976, p. 144)

What a formal language could offer, and what the Reed-Frost epidemic theory materialised in its mechanical analogue, was to "bring into view the different variables which may be concerned in determining the course of epidemics, to indicate in a general way how these may interact; and to call attention to the present gaps in our knowledge and imperfections in our means of observation."(Frost, 1976, p. 142) Importantly, this statement should not be misunderstood as a call for improved empirical measures. Instead, the model and its theory were intended to do nothing less but to define a new theoretical foundation for the epidemic as an open-ended object of research, characterised by interdependence and causal uncertainty. Rather than to define a general rate and scope of for the epidemic prevalence of a disease or to set arbitrary norms to separate endemic from epidemic states, Frost proposed to think of the epidemic as a temporary increase in prevalence, which indicates to the observer not more and not less than "a definite change in the balance of forces controlling the occurrence of the disease in the population."(Frost, 1976, p. 143) Rather than to conceptualize an epidemic through the consideration of cause and effect, Frost suggested in 1928 an epidemiological reasoning concerned with association and correlation; a thinking that is usually attributed to Austin Bradford Hill’s post-war work in non-communicable diseases (Berlivet, 2005).

Reed and Frost sought to emphasise interdependent variation instead of individual causes and to position epidemiology firmly as a science concerned with variable relations that controlled the waxing and waning of epidemic phenomena. With this definition, which Frost later also expanded to chronic disorders, epidemics ceased to be rare, exceptional events, but became phenomena characteristic of the fluctuations, oscillations and intervals stemming from the interplay between disease, environment and host population. The model achieved this iconoclasm through a subtle but crucial shift in focus. While most mathematical approaches to epidemics at the time had been predominantly concerned with the ratio of the population, Reed and Frost moved the focus of their mechanical model to the question of “adequate contact.” Their modelling thus substantiated an epidemiological reasoning, in which the cause for an epidemic could no longer be attributed to either the population, the pathogen or the environment but only to the conditions and structural forces that governed their interaction.

## Conclusion

As epidemiology became an academic department at Johns Hopkins, the field assumed a new identity as an analytical science. Proponents of epidemiology sought to establish the field as a science firmly oriented towards open-ended analysis of data from an equally open-ended range of data sources and to be applied to an increasingly open-ended range of phenomena. As the history of the Reed-Frost epidemic theory shows, this endeavour required a novel combination of descriptive and analytical methods, rather than the abolishment of empirical principles in the name of formalisation.

As the field of epidemiology was elevated to an academic discipline, epidemiologists began to invest in the production of their own, discrete theorization of epidemics as a research and as a working object. Turning epidemiological reasoning towards an analytical focus required the definition and representation of a research object in general terms and to subsume the study of increasingly complex infectious (and later chronic) diseases under one disciplinary umbrella. Where the epidemiologists' fieldwork observed the sheer infinite range of detail on the "epidemic streets", the epidemiologists’ lab-work and theoretical dedication now required a consideration of the proposed laws of epidemics through mechanistic models.

To understand the driver of a measles outbreak, for example, knowledge about household composition, rates of contact between children in a school or members of a community needed to be considered on the ground and within the local context. As Frost never grew tired to emphasize, this local configuration of an epidemic could not be approached, nor understood through models or mathematical abstraction, but demanded an approach in which observation and open-ended interest would prevail. Mathematical formalization and models, on the other hand, were not designed to define general laws with which one would understand the contingent space of a measles outbreak in a school. But models sought to provide students with a representation of the dynamics and relations that constitute the epidemic as an object of research to draw attention to the multiple, interdependent factors that drive any (measles) outbreak. In short, considering the model helped to prevent simplistic attributions for the outbreak to either a ‘weak’ population, or a particularly infectious virus, or just to specific sanitary conditions.

While some epidemiologists had begun to reconsider the interdependence between hosts and pathogens elsewhere, the mechanical analogue to the Reed-Frost epidemic theory offered a novel representation of the dynamics of epidemics as systems of balance and equilibrium. Thinking the epidemic through the model enabled a kind of agnosticism towards any causal theory, which were offered by physiology, eugenics, bacteriology or clinical medicine. Instead, the model diverted focus away from the identification of causality and established a system of thinking in which epidemics were neither a foreign invasion, nor an indication of sudden disruption. Instead, the model implied for an epidemic—even if it was associated with an identifiable infectious pathogen—to be merely a quantitative variation of otherwise normal circumstances. Perhaps most significantly, the mechanistic model also enabled the theorization of contagion beyond the concept of discernible pathogens. How and it what way such modelling supported the expansion of epidemiological reasoning into other domains and how it advanced the conceptualisation of contagious phenomena in sociology, economics and psychiatry, particularly in the post-war period, shall be subject of follow-on research.

Mechanistic modelling with SIR parameters has become the standard instrument to evaluate outbreaks and to predict potential disasters, particularly when data and observations are sparse. This history of the Reed-Frost epidemic theory draws attention to two aspects that are rarely considered when discussing the sudden authority and pervasive impact of modelling in the response to COVID-19. First, mechanistic modelling has for almost a hundred years determined how epidemiologists have theorized and analysed infectious diseases. The Reed-Frost epidemic theory has not assumed its position due to its capacity to accurately predict epidemic curves, nor due to its applicability to real-world outbreaks. Instead, the model’s pivotal position derives from instigating a shift in thinking, enabling the theorizing and conceptualising of epidemics “sui generis,” and by considering the dynamic conditions under which “adequate contact” between agent, environment and population might govern the dynamics of an epidemic outbreak. Second, this history emphasises the outstanding significance of such modelling in the modern history of epidemiology, and particularly in infectious disease research and education. However, this history also shows that models have not always been the intractable and opaque black boxes, as they appear today; their history as pedagogical instruments implies a shifting position of models and modelling in epidemiological practice since the 1920s. Questions need to be raised how a working object used to exemplify theoretical approaches in epidemiology has advanced into a fetishized point of convergence in the digital world, imbued with opaque academic hierarchies, shielded by technological boundaries but nonetheless equipped with authority over significant policy decisions.

## Footnotes

1 The SIR model is perhaps the most common compartmental modelling technique in contemporary infectious disease epidemiology. The letters describe the numbers of individuals who are either susceptible, infected or recovered and the model is widely referred to as the standard model for differential equations that seek to describe the dynamics of infectious diseases. (Lessler and Cummings 2016)

2 Frost, W.H. (1931), Frost Lecture on Epidemic Theory 1930, March 9^th^, 1931, Johns Hopkins University Archives, Wade Hampton Frost Papers, Box 60.

3 As neither Reed or Frost provided much of an explanation on the mathematical definitions of their model, this and the following inferences follow Helen Abbey’s interpretation of the mathematical theory of Reed and Frost (Abbey 1952).

4 The law of mass action was derived from the observation in chemistry concerning the role of reactants in equilibrium systems. Translated to epidemiology and ecology, Heesterbeck argues that it “roughly states that the rate at which individuals of two types, X and Y, meet is proportional to the product of the (spatial) densities of the respective subpopulations.” (Heesterbeek 2005)

5 Frost, W.H. (1931), Frost Lecture on Epidemic Theory 1930, March 9^th^, 1931, Johns Hopkins University Archives, Wade Hampton Frost Papers, Box 60.

6 The segment had been broadcasted on 17 April 1951, “Epidemic theory—what is it?”, Johns Hopkins Television Programs 1948–1960, Special Collections Milton S. Eisenhower Library, The Johns Hopkins University.

7 Frost, W.H. (1920), Frost's outline for Organization of a Department of Epidemiology, 1920, Johns Hopkins University Archives, Wade Hampton Frost Papers, Box 7.

8 Frost, W.H. (1925), Lecture Notes, Epidemiology I: 1925 Statistics, Johns Hopkins University Archives, Wade Hampton Frost Papers, Box 65, 1925–1930.

9 Frost, W.H. (1920), Frost's outline for Organization of a Department of Epidemiology, 1920, Johns Hopkins University Archives, Wade Hampton Frost Papers, Box 7.

10 Frost, W.H. (1920), Frost's outline for Organization of a Department of Epidemiology, 1920, Johns Hopkins University Archives, Wade Hampton Frost Papers, Box 7.

11 Frost, W.H. (1920), Frost's outline for Organization of a Department of Epidemiology, 1920, Johns Hopkins University Archives, Wade Hampton Frost Papers, Box 7.

12 Frost, W.H. (1931), Frost Lecture on Epidemic Theory 1930, March 9^th^, 1931, Johns Hopkins University Archives, Wade Hampton Frost Papers, Box 60.

13 Frost, W. H. (1927), Scope of Epidemiology, Conference on Epidemiology 1927, Johns Hopkins University Archives, Wade Hampton Frost Paper, Box 55.

14 As a testament to the popular current of the time this rationality most often led to eugenic propositions. Confronted with a dedication to the principles of social medicine among epidemiologists, Pearson and Greenwood failed to convince the Epidemiological Society in London of the need to seize their reformist public health activities, and to accept that hereditary rather than sanitary factors were supposedly behind recurring tuberculosis epidemics (Amsterdamska 2005, p. 26).
